# Comparison of three different application routes of butyrate to improve colonic anastomotic strength in rats

**DOI:** 10.1007/s00384-016-2718-z

**Published:** 2016-12-09

**Authors:** J. W. A. M. Bosmans, A. C. H. M. Jongen, B. T. C. Boonen, S. van Rijn, F. Scognamiglio, L. Stucchi, M. J. J. Gijbels, E. Marsich, N. D. Bouvy

**Affiliations:** 1 Department of General Surgery, Maastricht University Medical Center, P.O. Box 5800, 6202 AZ Maastricht, the Netherlands; 2 NUTRIM School for Nutrition and Translation Research in Metabolism, Maastricht University, Maastricht, the Netherlands; 3 Department of Life Sciences, University of Trieste, Trieste, Italy; 4 SIGEA Srl, AREA Science Park, Trieste, Italy; 5 Departments of Pathology and Molecular Genetics, CARIM, Maastricht University, Maastricht, The Netherlands; 6 Department of Medical Biochemistry, Academic Medical Center, Maastricht, the Netherlands; 7 Department of Medical, Surgical and Health Sciences, University of Trieste, Trieste, Italy

**Keywords:** Colorectal anastomotic leakage, Anastomoses, Animal study, Butyrate, Anastomotic healing

## Abstract

**Introduction:**

Despite extensive research, anastomotic leakage (AL) remains one of the most dreaded complications after colorectal surgery. Since butyrate enemas are known to enhance anastomotic healing, several administration routes have been explored in this study.

**Methods:**

Three intraluminal approaches involving butyrate were investigated: (1) butyrin-elucidating patch, (2) a single injection of hyaluronan-butyrate (HA-But) prior to construction of the proximal anastomosis and (3) rectal hyaluronan-butyrate (HA-But) enemas designed for distal anastomoses. The main outcome was AL and secondary outcomes were bursting pressure, histological analysis of the anastomosis, zymography to detect MMP activity and qPCR for gene expression of MMP2, MMP9, MUC2 and TFF3.

**Results:**

Neither the patches nor the injections led to a reduction of AL in experiments 1 and 2. In experiment 3, a significant reduction of AL was accomplished with the (HA-But) enema compared to the control group together with a higher bursting pressure. Histological analysis detected only an increased inflammation in experiment 2 in the hyaluronan injection group compared to the control group. No other differences were found regarding wound healing. Zymography identified a decreased proenzyme of MMP9 when HA-But was administered as a rectal enema. qPCR did not show any significant differences between groups in any experiment.

**Conclusion:**

Butyrate enemas are effective in the enhancement of colonic anastomosis. Enhanced butyrate-based approaches designed to reduce AL in animal models for both proximal and distal anastomoses were not more effective than were butyrate enemas alone. Further research should focus on how exogenous butyrate can improve anastomotic healing after gastrointestinal surgery.

**Electronic supplementary material:**

The online version of this article (doi:10.1007/s00384-016-2718-z) contains supplementary material, which is available to authorized users.

## Introduction

Despite extensive research, anastomotic leakage (AL) remains one of the most dreaded complications after colorectal surgery. It still results in high morbidity and mortality, and causes prolonged hospital stay and high healthcare costs [1–3]. Many studies have been performed regarding this subject, all aiming at improvement of anastomotic healing and thereby preventing leakage. Since the incidence of anastomotic leakage has not declined over the years, the need for a solution continues to exist [4, 5].

Previous animal studies have shown that rectal administration of butyrate—one of the predominant short chain fatty acids (SCFAs)—results in a higher bursting pressure in colonic anastomoses, thereby claiming an enhanced strength which may prevent anastomotic leakage [6–8].

These positive results did not change current practice, nor have human studies been reported in which patients received postoperative butyrate enemas to enhance anastomotic strength. This is not surprising, when you consider that clinicians are not keen on enemas directly after surgery, especially when a fragile rectal anastomosis is involved. Water-soluble contrast enemas are often used to determine the presence of colorectal AL, mostly after rectum resection. However, these enemas are never given in the very early postoperative phase (<postoperative day 5) due to the potential risk of complications [5]. Another disadvantage of the use of enemas is the low compliance rate and the short and discontinuous exposure of butyrate to the colonic mucosa.

A recent study however showed that butyrate has remarkable anti-inflammatory and regenerative effects improving the repair of intestinal mucosa in rats with colitis [9]. Butyrate stimulates re-epithelisation and may influence collagen lysis by reducing matrix metalloproteinase (MMP) release [10]. Butyrate is a product of the fermentation process by bacteria that occurs mainly in the distal colon. Because it serves as the most important energy source for colonocytes [11], our hypothesis is that increasing the concentration of butyrate will lead to an enhanced anastomotic healing in the colon. A number of studies indicate that butyrate affects the composition and thickness of the colonic mucus layer through alteration of mucin gene expression [12] and nutritional deficiency of the colonic epithelium and that butyrate also can act as a signalling molecule through specific G-protein-coupled receptors that are involved in the immune surveillance of the colonic mucosa towards microbial activity [13]. More recently, it has been shown that mice with an insufficient mucus layer on the colonic mucosa (Muc2^−/−^) suffered more often from anastomotic leakage compared to control mice [14].

Furthermore, animal studies showed that butyrate enemas have a significant positive effect on the left colonic anastomoses, but this effect was weaker on the right side of the colon [8]. Obviously, not the same volume will reach the proximal anastomotic site compared to the distal anastomosis, which may explain these findings. Another explanation might be that the butyrate-producing bacteria mainly reside in the mucus layer at the distal part of the colon, that the proximal colon does not respond to a higher amount of butyrate, simply because the proximal enterocytes are not familiar to having that energy source available and are not able to use it. To increase the beneficial effect of butyrate, we added hyaluronan (HA), a glycosaminoglycan that is known to promote neovascularization, to enhance the process of scarring, and it has beneficial effects on cell proliferation in several tissues [15, 16]. Hyaluronan also plays a role in the downregulation of the inflammatory response. Its free-radical scavenging and antioxidant properties and its supposed inhibiting effect on proteinases such as MMPs seem to be responsible for this downregulation, stabilizing the granulation tissue during the healing process [16].

In this study, we investigated several ways to improve the proximal anastomotic strength by applying butyrate near this anastomosis: using a patch, injections and enemas. Our aim was to develop a method that resulted in a lower anastomotic leakage rate and more practical methods that can be applied in daily clinical practice.

## Methods

### Animals

Eighty-four Wistar rats with a body weight of 250–300 g were used. Animals were housed and cared for at the Central Animal Facility of Maastricht University. All animals were provided ad libitum access to food and water and were cared for according to local standards. Postoperatively, welfare assessment was performed twice daily using a standardized method and animals were given pain medication in case of discomfort. The experimental protocol complied with the Dutch Animal Experimental Act and was approved by the Animal Experimental Committee of Maastricht University Medical Center. The ARRIVE guidelines for reporting in vivo experiments were followed [17] (see also supplementary data S1).

### Study design

Primary objective of the study is to investigate if anastomotic leakage can be reduced in a leakage model by application of either an intraluminal butyrin-eluting patch, a single-time intraluminal administration of butyrate or a daily HA-But enema. For the proximal anastomoses, we used both an intraluminal butyrin-eluting patch that covered the anastomosis and a single-time intraluminal administration of hyaluronan-butyrate (HA-But). Since most research is performed in the distal part of the colon, we also used a left-colon anastomosis model to test the HA-But enemas. This resulted in the following three experiments:

1. Butyrin-eluting patches (20 massa% tributyrin) versus placebo patches (*n* = 12/group)
2. HA-But injection (5% HA, 60 mM butyrate) versus HA injection (5% HA) versus saline injection (0.9% NaCl) (*n* = 8/group)
3. Five millilitres of HA-But enema (5% HA, 60 mM butyrate) versus 5 mL sodium butyrate (60 mM) enema versus a control group that received no enemas (*n* = 12/group)

Patches were kindly provided by Polyganics (Groningen, the Netherlands), and enemas and injections were produced by Sigea (Trieste, Italy).

In all experiments, follow up was 7 days.

### Surgical procedure

All rats received 0.05 mg/kg buprenorphine as analgesic and were anaesthetised using isoflurane. To acquire access to the abdominal cavity, a 5-cm craniocaudal midline incision of the skin and abdominal musculature was made in all experiments. The cecum was then identified and moved outside of the peritoneal cavity and onto sterile gauzes that were hydrated with sterile saline solution to prevent dehydration. For proximal anastomoses, the colon was transected two centimetres distal from the cecum and an end-to-end anastomosis was created using four interrupted polypropylene 6/0 sutures (Prolene, Ethicon, Johnson & Johnson) (Exp 1& 2, see supplementary data S1). In experiment 3, the anastomosis was performed in similar fashion but at 4 cm ab ani. After performing the anastomosis, the intestines were repositioned and the abdomen was closed in two layers, a running suture for the muscle layer (Vicryl 4-0, Ethicon, Inc) and interrupted sutures for the skin (Monocryl 4-0, Ethicon, Inc).

### Macroscopic endpoints

Anastomotic leakage was defined as (1) no anastomotic leakage, (2) small abscess at the anastomotic site <1cm^3^, (3) large (>1cm^3^) abscess at the anastomotic site or (4) complete dehiscence with peritonitis/death due to faecal peritonitis. Adhesions to the anastomotic site were assessed according to van der Ham et al. [18]

### Bursting pressure

Bursting pressure was measured as previously described [19]. In short, a 5-cm segment of intestine including the anastomosis with adherent organs was resected en bloc and the part distal of the anastomosis was clamped. A plastic tube was inserted in the proximal end and ligated with a single polyglactine 4/0 suture (Vicryl, Ethicon). The complete anastomosis was immersed in phosphate-buffered saline, while air was infused using a manometer (IDEE, Maastricht University, the Netherlands) and pressure was manually increased by inflating the colon. Bursting pressure was defined as the intraluminal pressure at which air leakage was initially observed from the anastomosis (mBar).

### Tissue preparation

In anaesthetised rats, the anastomotic site was dissected (after measuring bursting pressure) with a 0.5-cm margin at each site of the anastomosis. Tissue samples were divided in three equal pieces: one for qPCR, one for zymography purposes and one for immunohistochemistry purposes. This latter one was cut in longitudinal direction and tissue was stretched and pinned onto a cork layer in order to secure a straight anastomotic line and improve quality of histological assessment prior to fixation in formalin (supplementary data S2). Sections were deparaffinized in xylene and rehydrated in graded ethanol to distilled water prior to performing histological staining. The part dedicated for qPCR analysis was snap frozen in liquid nitrogen and stored at −80 °C until required for mRNA isolation.

### Histology and immunohistochemistry

Sections were stained with standard haematoxylin-eosin and Picro Sirius Red using Direct Red 80 and Picric Acid solution (both Sigma-Aldrich, the Netherlands).

An independent, experienced animal pathologist performed blinded histological assessment on the obtained tissue, scoring (from 0 to 4) histological sections on inflammation, granulocyte influx, fibroblast activity and collagen deposition. Collagen ration was calculated using an in-house software program on pictures obtained with a polarized light microscope (Leica DM5000B, Leica Microsystems, Wetzlar, Germany).

### MMP2, pro-MMP9 and MMP9 activity analysis using zymography

MMP2 and MMP9 activities were assessed using gelatin zymography since its increase of MMP activity has been implicated in the pathogenesis of AL [20]. Samples were lysed from tissue and loaded on a polyacrylamide gel containing gelatin (10% Ready Zymogram Gel, Bio-Rad, the Netherlands). Gel electrophoresis took place at 100 V for 30 min. After that, the gel was incubated with 2.5% Triton X-100 in MQ for 30 min at room temperature. The gel was then placed in developing buffer at 37 **°**C overnight. Colouring of the gel took place the following night with PAGE-Blue protein staining solution (Thermo Scientific, the Netherlands). At day 3, the staining was decoloured with MQ, revealing MMP2 and MMP9 activity. The gels were scanned using a Bio-Rad GS-800 Densitometer and digital quantification was done using ImageJ software (ImageJ Software, U.S. National Institutes of Health, Bethesda, Maryland, USA).

### MMP2, MMP9, TFF3 and Muc2 expression analysis using qPCR

The expression of matrix metalloproteinases 2 (MMP2) and 9 (MMP9) as well as mucin 2 (MUC2) and trefoil factor 3 (TFF3) was determined using qPCR to see if exogenous butyrate had an effect on the expression of these MMPs and the abundant secretory mucin MUC2 as well as its associated trefoil factor. All qPCR-experiments were carried out by a dedicated technician (BB) and are reported as detailed as possible according to the MIQE guidelines [21]. RNA was isolated from the tissue sample using TRI reagent (Sigma-Aldrich, the Netherlands) and treated with RNase-Free DNase Set (Qiagen). The purity of RNA was tested using Nanodrop Spectrophotometer (Thermo Scientific) by 260/280 absorbance (A260/A280) ratios, ranging from 1.89 to 2.02. Therefore, the purity of the isolated RNA was satisfactory for further studies. RNA of 750 ng was converted to DNA with sensifast cDNA Synthese kit (Bioline, London, UK). qPCR was performed on 10 ng cDNA with 0.3 μM primers in Sensimix SYBR & Fluorescein Kit (Bioline, London, UK) using white 384-wells qPCR plate. Primer sequences are listed in Table 1. Reference genes included were glyceraldehyde-3-phosphate dehydrogenase (GAPDH) and B-actin. Reactions were run on the LightCycler480 (Roche). PCR conditions used were 10 min at 95 °C, followed by 40 amplification cycles of 15 s at 95°-60 °C–72 °C followed by a melting curve to validate the amplifications. The cycle threshold (Ct) value of each reference gene was obtained from qPCR analysis. The obtained Ct values were applied to evaluate the expression of the genes of interest. The mean Ct value of the reference genes ranged from 16 to 23 cycles, with HA-injection having the highest transcript levels in MMP9, and butyrin patch was associated with having the lowest transcript levels in TTF3. For analysis purposes, the level of MMP2 expression of the saline group was set at 1 and used as a control.

**Table 1:** Primer sequences used for qPCR experiments

r-MMP9-F1	AGCCGACGTCACTGTAACTG
r-MMP9-R1	AACAGGCTGTACCCTTGGTC
r-MMP2-F1	ACAACAGCTGTACCACCGAG
r-MMP2-R1	GGACATAGCAGTCTCTGGGC
R-MUC-F1	CGAAGTGAAGAGTGAGCACG
R-MUC-R1	GATCCGGGTGGTATTCAGCA
R-Tff-F1	GGCCTATCTCCAAGCCAATGT
R-Tff-R1	TGCAGAGGTTTGAAGCACCA
GAPDH FW	GGAAGCTCACTGGCATGGC
GAPDH RV	CCTGCTTCACCACCTTCTTG
Beta-actin FW	GACAGGATGCAGAAGGAGATTACTG
Beta-actin RV	CCACCGATCCACACAGAGTACTT

### Statistical analyses

Statistical analysis was performed using Prism 5.0 for Mac (Graphpad software, San Diego, CA). Normality was tested using Kolmogorov-Smirnov. All continuous variables are presented as mean and standard error of the mean (SEM) and compared using student’s *t* test or Mann Whitney U when appropriate. Dichotomous variables were compared using the Chi-square test.

## Results

### Macroscopical results

**Fig. 1:**
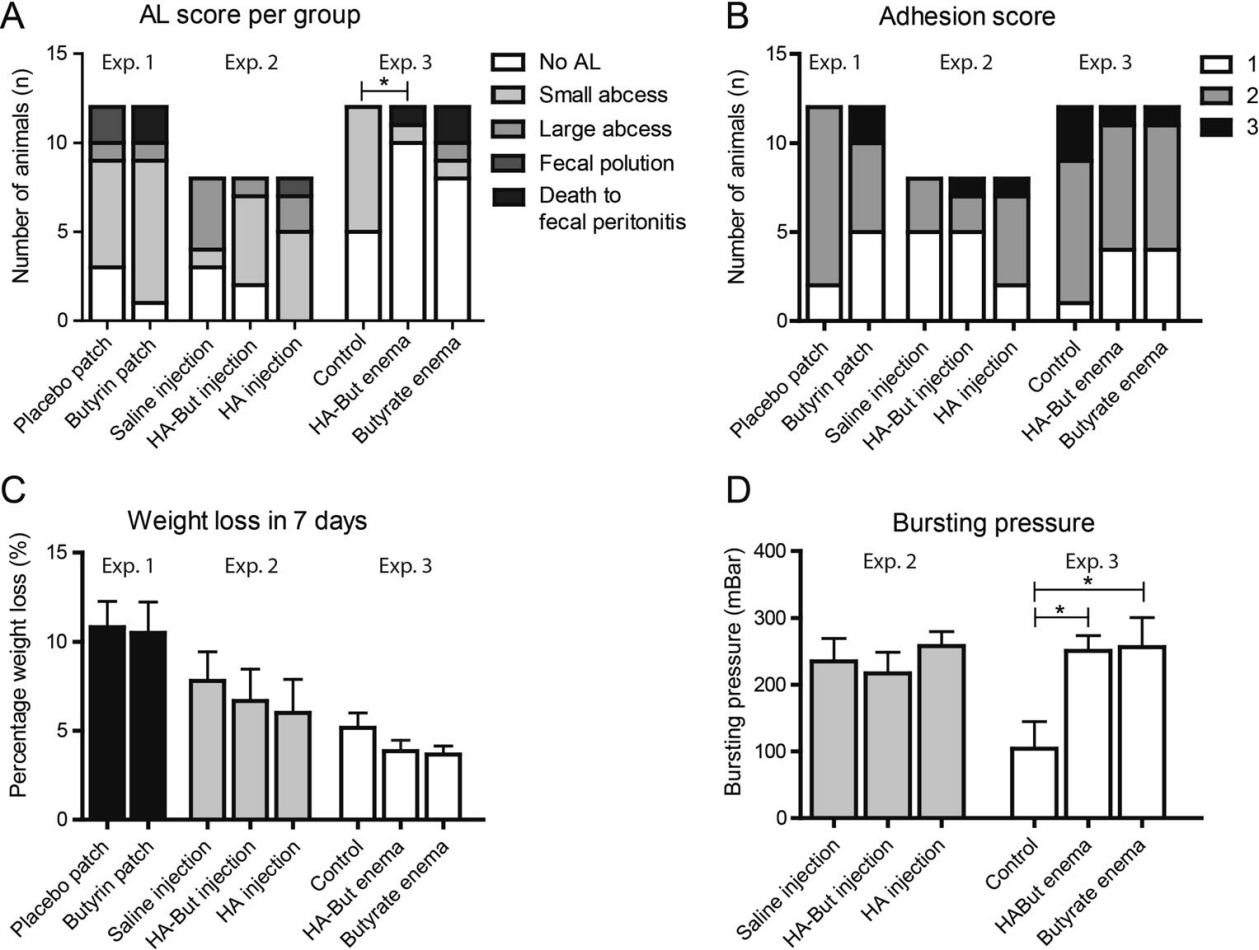
Butyrate-enriched enemas reduced AL in Exp. 3, no other butyrate-based intervention caused a decrease in AL rates (**a**). Adhesion scores did not differ significantly between groups in Exp. 1, 2 or 3 (**b**). Weight loss was most prominent in Exp. 1 where placement of the patches caused a sudden change in calibre of the colon; animals receiving enemas had the lowest percentage of weight loss but also received a different type of anastomosis compared to animals in Exp. 1 and 2 (distal vs proximal respectively) (**c**). The administration of butyrate enemas caused a significant higher bursting pressure of the anastomosis in Exp. 3 compared to the control group. No differences were found in Exp. 2 and in Exp. 1; no BP was measured due to porous, distended intestines

In experiment 1, two rats in the butyrin patch group died prior to completing follow-up due to faecal peritonitis. All animals in experiment 2 completed the 7-day follow up. In experiment 3, three rats (one in the HA-But group, three in the But group) were euthanized because they had reached humane endpoints, due to faecal peritonitis. An overview of the types of anastomotic leakage within each experimental group can be found in Fig. 1a. Adhesions occurred in every experiment, and there were no significant differences between experiments (proximal versus distal anastomoses) nor within each experiment (Fig. 1b). It became evident that animals receiving a patch (either butyrin eluting or placebo) had lower welfare scores and significant more weight loss compared to animals receiving enemas (Fig. 1C), possibly due to an extended bowel proximal to the location of the patch upon sacrifice.

### Bursting pressure

Bursting pressure (BP) was not measured during experiment 1 because of evident extended intestines in the majority of the rats at the place where the patches were placed. This abrupt change in luminal calibre made the dilated bowel very porous, disabling accurate BP measurements. In experiment 2, BP was measured and compared to a historical control group since the control group from experiment 3 received a distal anastomosis. No differences were found in experiment 2 between saline injections, HA injections and HA-But injections. In experiment 3, however, a significant difference could be found between the control group (104.1 ± 40.8) and HA-But (250.7 ± 23.1) and But enemas (256.4 ± 44.4, *p* < 0.05).

### Histology and collagen ratio

**Fig. 2:**
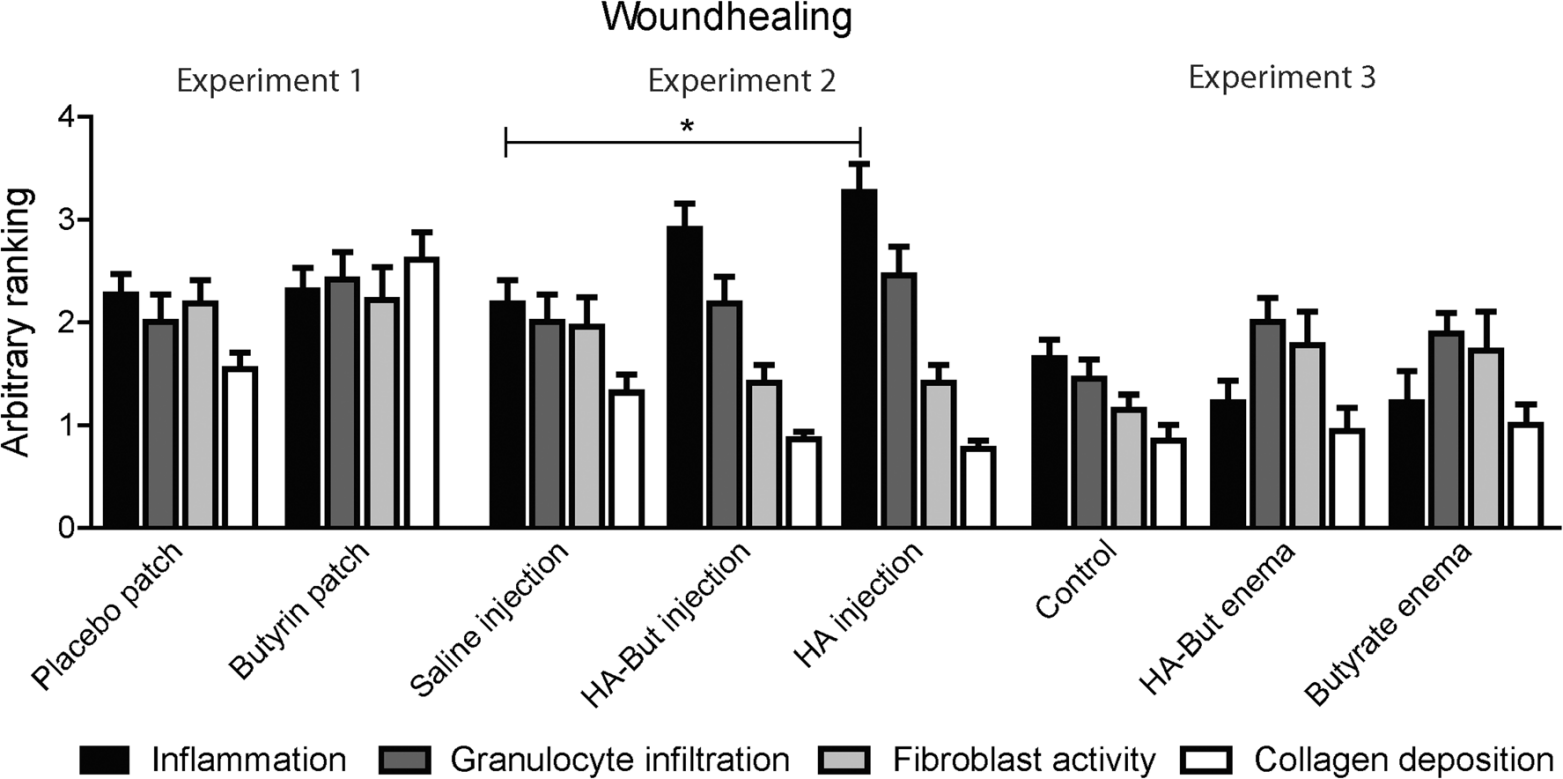
Histological analysis only detected an increased inflammation in experiment 2 in the hyaluronan injection group (2.18 ± 0.22) compared to the control group (3.27 ± 0.27, *p* < 0.05). No other differences were found regarding wound healing. Values are mean ranking (S.E.M.)

Inflammatory parameters were scored as a surrogate marker of anastomotic healing, as indicated appropriately by a recent Delphi consensus method [22]. Histological analysis (Fig. 2) only detected an increased inflammation in experiment 2 in the hyaluronan injection group (2.18 ± 0.22) compared to the control group (3.27 ± 0.27, *p* < 0.05). No other differences were found regarding wound healing. Picro Sirius red staining, which depicts the level of collagen maturity, of the anastomotic region showed comparable percentages of collagen for all groups (Table 2).

**Table 2:** Total collagen and mature/immature collagen ratios

Experiment 1	Butyrin patch	Placebo patch	*p* value	
Collagen percentage (%)	32.25 (±2.69)	28.64 (±2.00)	0.239	
Red/green ratio	464.83 (±278.36)	361.42 (±207,96)	0.861	
Experiment 2	Saline injection	HA-But injection	HA injection	*p* value
Collagen percentage (%)	53.22 (±2.04)	45.29 (±4.51)	46,50 (±4.83)	0.361
Red/green ratio	66.89 (±16.76)	39.66 (±7.88)	95.64 (±44.43)	0.340
*Experiment 3*	*Control*	*HA-But enema*	*Butyrate enema*	*p value*
Collagen percentage (%)	42.84 (± 4.75)	35.57 (± 4.23)	35.93 (± 2.12)	0.343
Red/green ratio	321.50 (±71.60)	282.00(±154.85)	176.54 (±46.35)	0.355

The relative collagen area was quantified as the percentage of total tissue surface. Maturity of collagen was estimated by calculating the red/green ratio. Red indicates thick, mature collagen. Green indicates thin, immature collagen In terms of collagen percentage and red/green ratio, no significant differences were found between groups. All measurements in this table are presented as mean (±SEM)

### Pro-MMP9, MMP2 and MMP9 activities measured by zymography

**Fig. 3:**
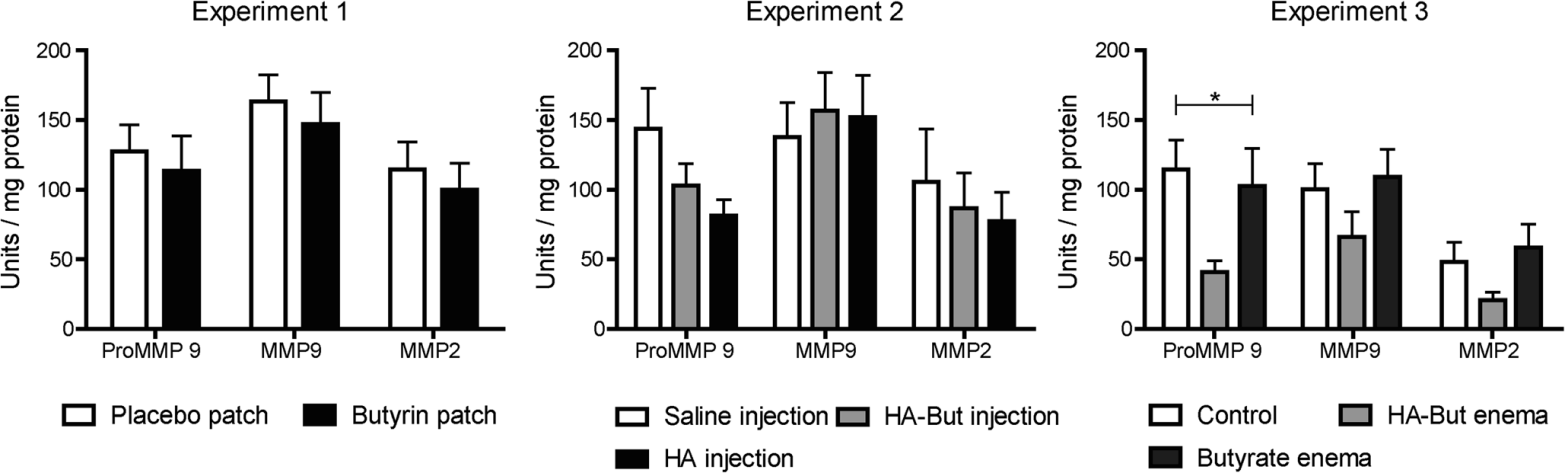
Zymography revealed no differences between MMP2 and MMP9 activity within each experiment between control and intervention groups. The addition of hyaluronan to the butyrate enema did decrease the activity of pro-MMP9 (40.6 ± 8.2) compared to the butyrate enema alone (102.6 ± 26.9) and the control group (114 ± 20.9) in experiment 3 (*p* = 0.01). Values are given in arbitrary units and data represent mean + S.E.M.

Zymography revealed no differences between MMP2 and MMP9 activity within each experiment between control and intervention groups (Fig. 3). There was relatively large variability within the samples of each group as can be seen by the error bars/whiskers in the different graphs. In experiment 3, the addition of hyaluronan to the butyrate enema did decrease the activity of pro-MMP9 (40.6 ± 8.2) compared to the butyrate enema alone (102.6 ± 26.9) and the control group (114 ± 20.9, *p* = 0.01).

### qPCR showed no significant differences in the three experiments regarding gene expression

**Fig. 4:**
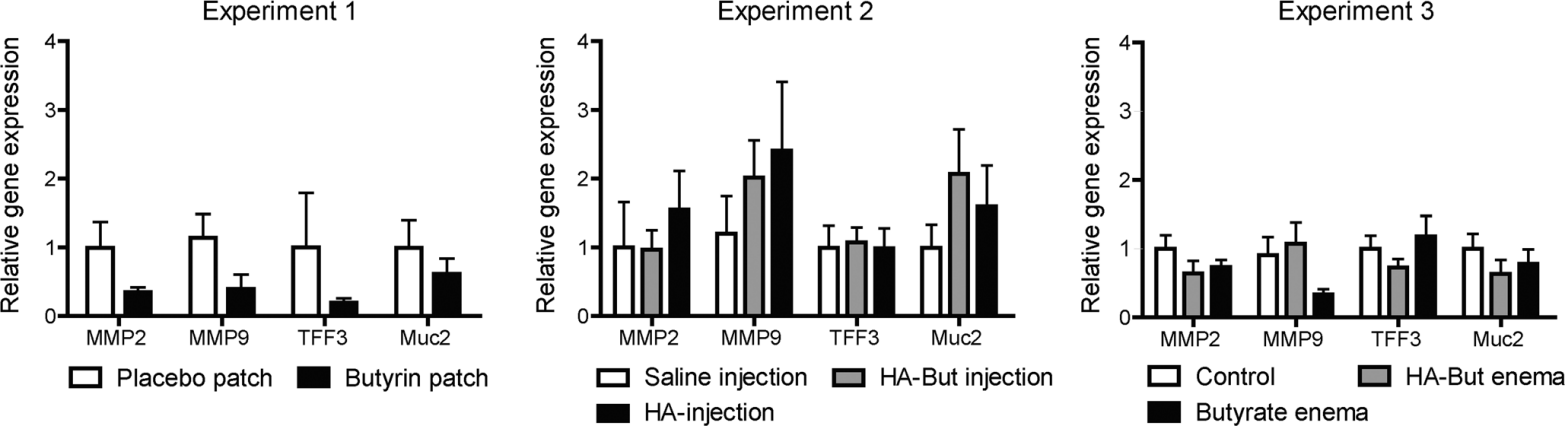
Overview of relative gene expression where the level of MMP2 expression of the saline group was set at 1 and used as a control. No significant differences were found in gene expression between the intervention and control groups regarding all genes of interest in the separate experiments

In experiment 1, a trend was found in the expression of MMP2 between the group that received a butyrin patch (0.35 ± 44.4) and the placebo patch group (1.14 ± 0.34, *p* < 0.07, Fig. 4). The other tested matrix-metalloproteinase MMP9 also showed a trend between these two groups, 0.39 ± 0.21 versus 1.14 ± 0.34 respectively, *p* < 0.08. No differences were found regarding MUC2 and TFF3. In experiments 2 and 3, no significant differences were found in gene expression between the intervention and control groups regarding all genes of interest.

## Discussion

This study is the first study as far as we know that aims to improve butyrate supplementation in animal models to overcome the reservation of surgeons to optimize their patients with butyrate prior or during gastrointestinal surgery. We investigated different administration routes besides enemas that could enable surgeons to incorporate butyrate in the surgical procedure. Unfortunately, a single dosage of butyrate or a butyrin-eluting patch that we used in this study did not seem promising. Addition of hyaluronan to butyrate did not improve the effect of an enema on the healing of distal anastomoses. Unfortunately, this study did not lead to promising tools to transport butyrate to the proximal anastomotic site, but it did confirm that butyrate can lead to better anastomotic healing and this should be further investigated in the future.

Butyrate is known to have a critical mediating role in the colonic inflammatory response; for example, it can induce the differentiation of colonic regulatory T cells and thereby ameliorate the development of colitis as shown by Furusawa et al. [23]. More recently, it has been shown that exogenous butyrate restoration improved intestinal epithelial cell junctional integrity, decreased apoptosis and mitigated graft-versus-host-disease after allogeneic bone marrow transplant [24]. Especially the restorative effect of butyrate on the epithelial integrity and, consequently, a decreased translocation of luminal contents are of great interest in the field of anastomotic leakage. It was often assumed that butyrate increases collagen synthesis and maturation and that this reduced the risk of anastomotic leakage [6–8]; however, with these new insights, it can be hypothesised that increased epithelial integrity leads to a lesser amount of (clinically relevant) anastomotic leakage.

As stated before, enemas seem to be a promising tool to deliver butyrate in the colon, especially in more distal anastomoses; however, it remains difficult to reach the same levels of butyrate in proximal anastomoses. In addition, butyrate is metabolized rapidly as soon as it enters the colonocyte via its active transport system. This could be the reason that experiment 2 was not successful; the amount of available butyrate by a single injection was simply not sufficient to cause any effect on the anastomotic site.

It was suggested that a prodrug of natural butyrate, tributyrin, could overcome the pharmacokinetic drawbacks of natural butyrate as a drug [25]. Since it was very difficult to incorporate butyrate in an eluting patch, butyrin was used instead, making sure that there were high concentrations available at the site of release. Unfortunately, the attachment of a patch into the colon caused extension of the bowel proximal to the location of the patch, indicating colonic stenosis. This effect of patches or fleeces has been previously described [26]; however, in that study, the authors used an external fleece and in experiment 1, the film-like resorbable patches were attached intraluminally. This stenosis effect—clinically translatable to ileus—could be the explanation of why the animals in experiment 1 had lower welfare scores and lower weight compared to the other groups.

Hyaluronan is generally known to be associated with tissue repair and wound healing, and its concentration is high in healing tissues [27]. Its biological functions are the result of both its physicochemical properties and its biological interactions, depending on molecular weight [28]. Despite the logical hypothesis that the addition of hyaluronan to butyrate would enhance anastomotic healing, this was not observed in this study, possibly due to a low concentration or not having the appropriate molecular weight.

One of the limitations of this study—even if the newly tested approaches were successful—is the translation to the human setting. While butyrate can be administered as a rectal enema, this is rather invasive and not the preferred choice in drug administration. The risk of anastomotic dehiscence in a recent constructed anastomosis remains, which could be an explanation of the faecal peritonitis that was found in the enema groups in experiment 3. However, the very unpleasant odour and taste of butyrate makes oral administration extremely difficult. For this reason, Raso et al. have studied a derivative of butyrate, N-(1-carbamoyl-2-phenyl-ethyl) butiramide (FBA) as a successful therapeutic alternative to butyrate, sharing a comparable efficacy, but a better palatability and compliance, unfortunately so far only in a rat model [29]. Other articles that describe oral administration of butyrate were also designed as animal studies [30, 31]. Nevertheless, there are a few studies in which oral butyrate or its prodrug was given in humans and was considered safe and well tolerated [32]. The question remains if butyrate can be administered as a local agent at the site of the anastomosis, without potential harmful effects when it enters the systemic circulation. Van Beek et al. have recently demonstrated that splanchnic butyrate release was prevented in patients after colonic butyrate administration, indicating that rectal enemas even with high therapeutic dosage (up to 100 mmol/L) of butyrate can be given safely [33].

Conceivably new approaches may become available as nano-based drug delivery systems for encapsulation and release of drugs are currently being investigated in different fields of medicine [34, 35]. Imaginably in the future, butyrate tablets will become standard perioperative treatment and due to nanotechnology, the release of butyrate will only take place at the site of interest, the colonic anastomosis.

In conclusion, butyrate can enhance the colonic anastomosis, making it less prone to lead to leakage. The most effective method thus far is by rectal enemas and the addition of hyaluronan did not ameliorate the effect of butyrate on the AL rate. Further research should be done to identify the optimal way of butyrate delivery to the anastomotic site where it can enhance anastomotic healing.

## Electronic supplementary material

ESM 1 (DOCX 58581 kb)
