# Supplementary material for: Comparison of three different application routes of butyrate to improve colonic anastomotic strength in rats
Source: Int J Colorectal Dis. 2016 Dec 9;32(3):305–13. doi: 10.1007/s00384-016-2718-z (PMC5316396; doi:10.1007/s00384-016-2718-z)
Supplement: Supplementary file 1 — (DOCX 58581 kb) [file 384_2016_2718_MOESM1_ESM.docx]

**S1: Supplementary information according to ARRIVE guidelines**

*Ethical statement*

- The experimental protocol complied with the Dutch Animal Experimental Act and was approved by the Animal Experimental Committee of Maastricht University Medical Center. Protocols for institutional animal use and care guidelines were followed (permit DEC 2013-101)

*Study design*

- In total there were 84 rats used in this study. We used 8 or 12 rats per experimental group, and 2-3 groups per experiment, as is shown in the table below:

| Experiment | Intervention | Control group | Number of animals | Proximal/ Distal colon |
| --- | --- | --- | --- | --- |
| 1 | Butyrin-eluting patch | Placebo patch | 12/group 24 in total | Proximal |
| 2 | Hyaluronan-butyrate injection | Hyaluronan injection and saline injection | 8/group  24 in total | Proximal |
| 3 | Hyaluronan-butyrate enema | Butyrate enema and no enema | 12/group 36 in total | Distal |

*Experimental procedures*

- For surgical procedure, see manuscript. This was carried out in the rat operating room of the animal facility of Maastricht University under semi-sterile conditions.


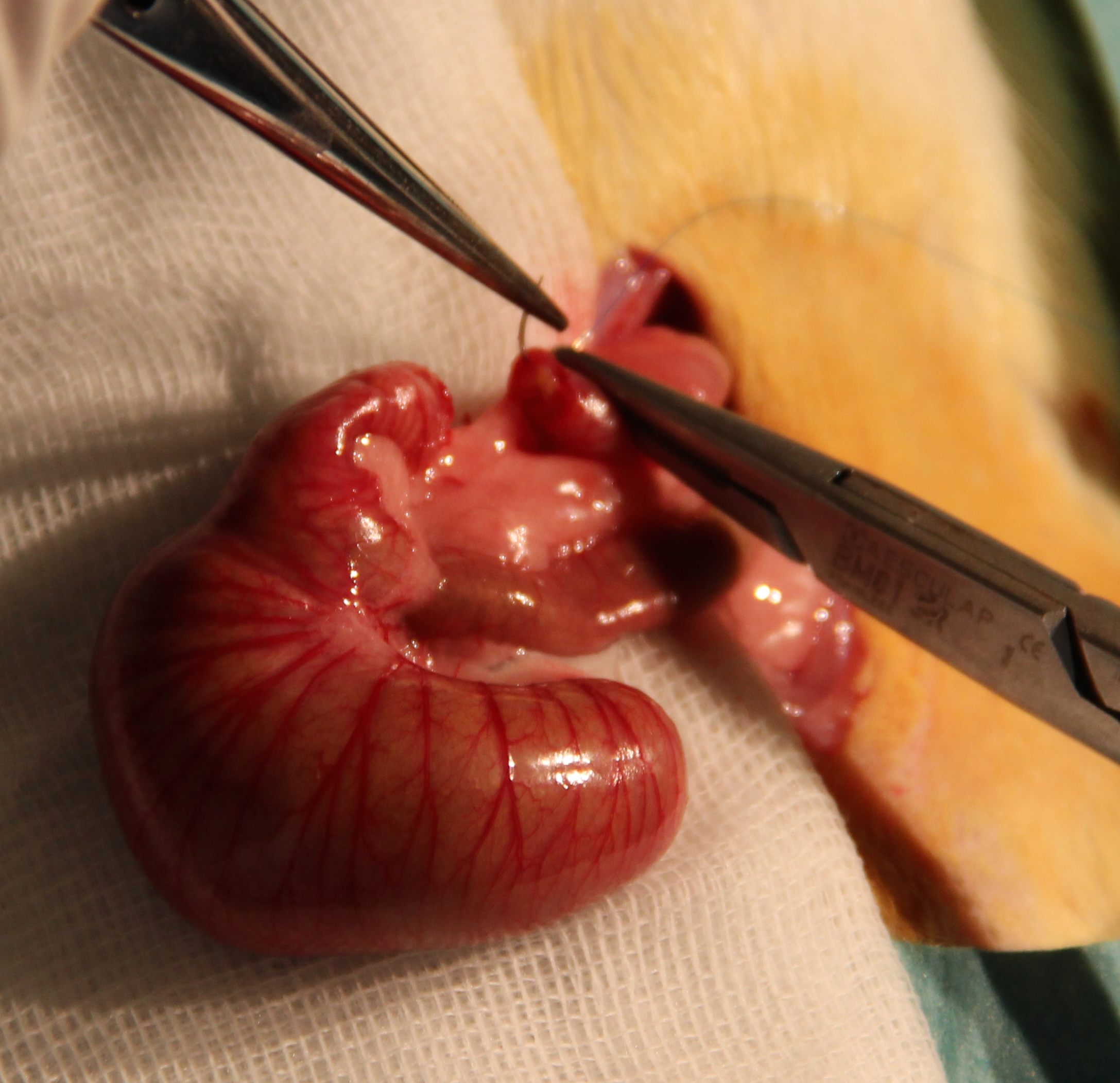

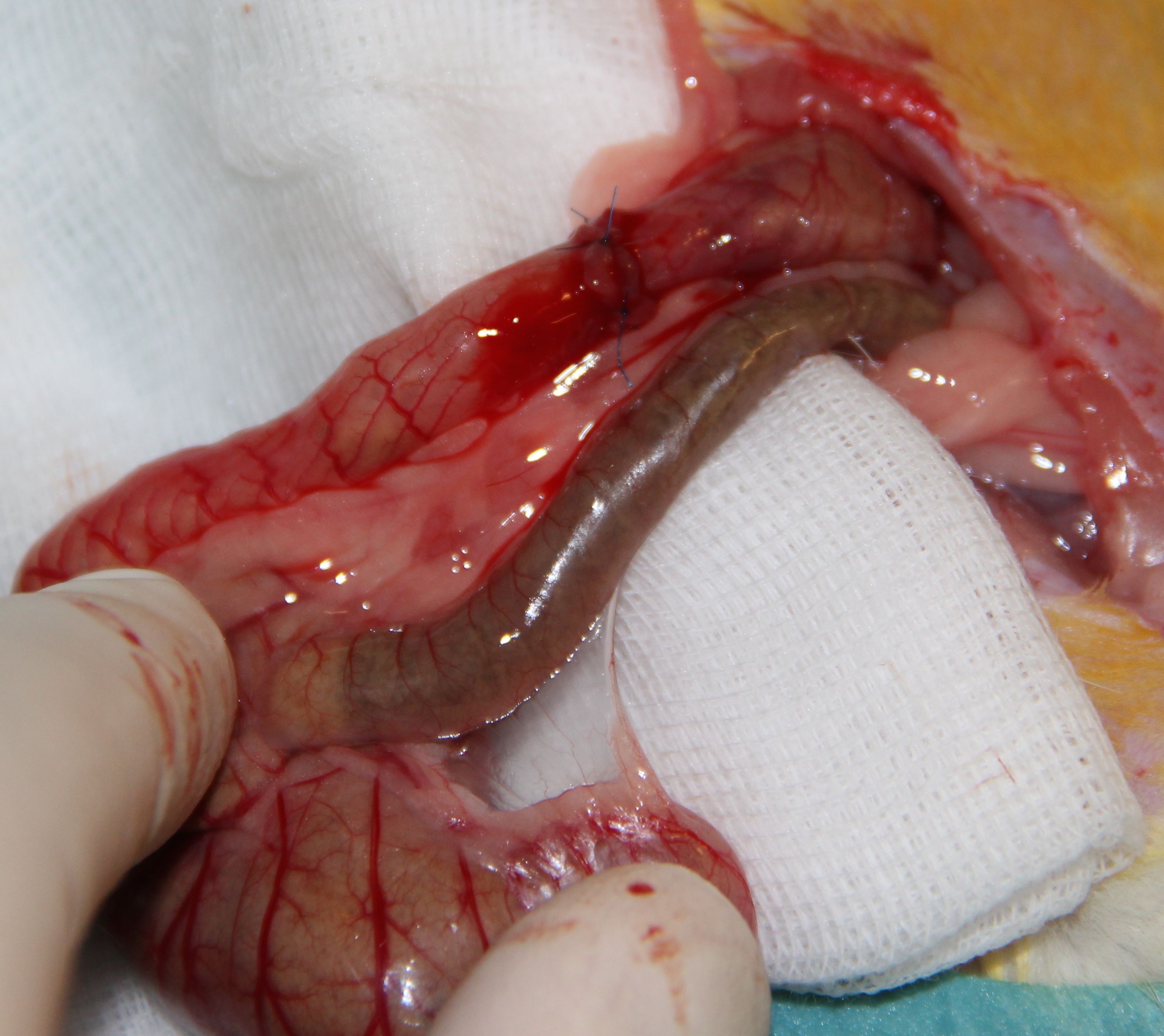


A

B

*Figure S1: Creating the proximal colonic anastomosis after transection (A), constructed using 4 interrupted sutures (B)*

- The enemas in experiment 3 were given on a daily basis, starting on postoperative day and continued until day 7 when rats were euthanized. The enemas were administered with the rat in a head-down position, without anesthesia. A cannula was inserted in the rectum and the enema was given by infusion. Afterward, rats were held in the head-down position for 30 seconds to ensure equal distribution of the liquid.

*Experimental animals*

- Male Wistar rats (RccHan:WIST) were purchased from Harlan laboratories, the Netherlands with a weight of 275g. Rats had an average weight of 327g on the day of surgery (SD 19.3).
- We have chosen for male rats, since it is known that female estrogens and androgens have an important influence on wound-healing (Ashcroft GS, et al. Estrogen modulates cutaneous wound healing by downregulating macrophage migration inhibitory factor. *The Journal of clinical investigation.* 2003, 111:1309-1318.)
- Since we value the 3R principle, we always try to reduce the amount of animals needed for experiments as much as possible. Since we have a lot of experience with proximal anastomoses in rodents, we have a lot of data available on this subject. It is known that Study design–related parameters such as laboratory, species/strain, route of administration, vehicle, feed, feeding practices, study duration, and housing have a potential to impact study outcomes and control findings. We have considered these parameters when selecting the appropriate studies for the historical control data. Although the use of historical control data from rodents is familiar in toxicology studies (Toxicol Pathol August 2009 vol. 37 no. 5 679-693), for efficacy studies is still remains uncommon, while it is scientifically valuable.

*Housing and husbandry*

- An acclimatization period of one week was observed prior to the start of the experiment.
- Rats were kept under standard conditions and were provided with food and water ad libitum. Rats were housed 2 animals per cage. The general health of rats was monitored several times per week for signs of inflammation and animals were weighed once per week. During the experiment animals were weighed daily and scored for discomfort twice daily (every morning and every evening). In case of discomfort, additional pain treatment was administered by giving buprenorphine 0.05mg/kg s.c.
- Discomfort was scored using a standard scheme (zie below). Humane endpoints were defined according to Roughan & Flecknell (*Roughan, J. V. & Flecknell, P. A. Behavioural effects of laparotomy and analgesic effects of ketoprofen and carprofen in rats. Pain 90, 65–74 (2001).*
- Humane endpoints are defined as:
  - Significant weight loss >20%
  - Fever (temperature is only measured on indication)
  - Tachypnoe
  - Significant differences in behavior:
    - Lethargia
    - Twitching: random spasms of the muscles, can be seen when animals are asleep or inactive in huddled up position.
    - Walking: unable to stand on four legs, wobbly walk
    - Huddled up posture: showing a concave abdominal side. Can be seen when walking/sitting.
  - Signs of severe dehydration
  - Severe diarrhea
  - Severely inflammed surgical wound
  - Cachexia

*Allocating animals to experimental groups*

- Animals were randomized by means of taking a card out of an envelope on which either 1-2-3 was printed. For each animal a card was drawn and the rat received the assigned treatment. We had 3 separate envelopes for each experiment a new envelope (with either 2 or 3 types of card in there).

*Numbers analysed*

- All analyses were performed according to an intention-to-treat analysis. However, since there were adverse events and deaths prior to follow-up, for ABP and histological assessment these animals were not taken into account.

**Welfare scoring rat model**

| **Description** | | | | |
| --- | --- | --- | --- | --- |
|  | **0** | **1** | **2** | **3** |
| **Activity** | Normal | Isolated, less active | Inactive | Somnulent, stupor, coma, lifeless |
| **Behavior** | Normal | Back arching, twitching, shivering  Once/10 min | Back arching, twitching, shivering | Stereotype behavior, auto mutilation, aggressive behavior |
| **Gait** | Normal | Mildly uncoordinated/  Abnormality | uncordinated walking on toes, limping | Paralysis, limp, convulsions, tremor. |
| **Posture** | Normal | Huddled up, stretching | Imbalance, twitching | Fall over, circle |
| **Physical condition** | Normal | BC2= condition | BC5= obese | BC1= emaciated BC6=extreme obese |
| **Fur/skin** | Normal | Dry, rough, not shiny anymore | Piloerection, small wounds, porfyrie, dry white skin | Red/black skin, inflammation, wounds, loss of fur |
| **Hydratation** | Normal | Loss of skin elasticity | Reduced skin turgor | Severly reduced turgor + sunken eyes |
| **Breathing** | Normal | Fast and superficial | Fast abdominal breathing + audible breathing | Respiratory problems, cyanosis, breathing with open mouth |
| **Faeces/urine** | Normal | Moist faeces, polyurie | Diarrhea, abnormal urine | Uncontrolled diarree, bloody stool, obstipation, hematuria |
| **Surgical wound** | Normal healing | Sutures intact, slighty red/bloody | Dehiscence of wound, sutures open, fluid secretion | Severe bleeding, wound open, severe redness, necrosis |
| **Edema** | Normal | Mild abnormal fluid collections, swollen appearance | Abnormal large abnormal fluid collections, ascites | Severe large abnormal fluid collections |
| **Necrosis** | Normal | Dark skin colouring | Small dark/black spots, burning wounds, blisters | Big black spots, crusts |


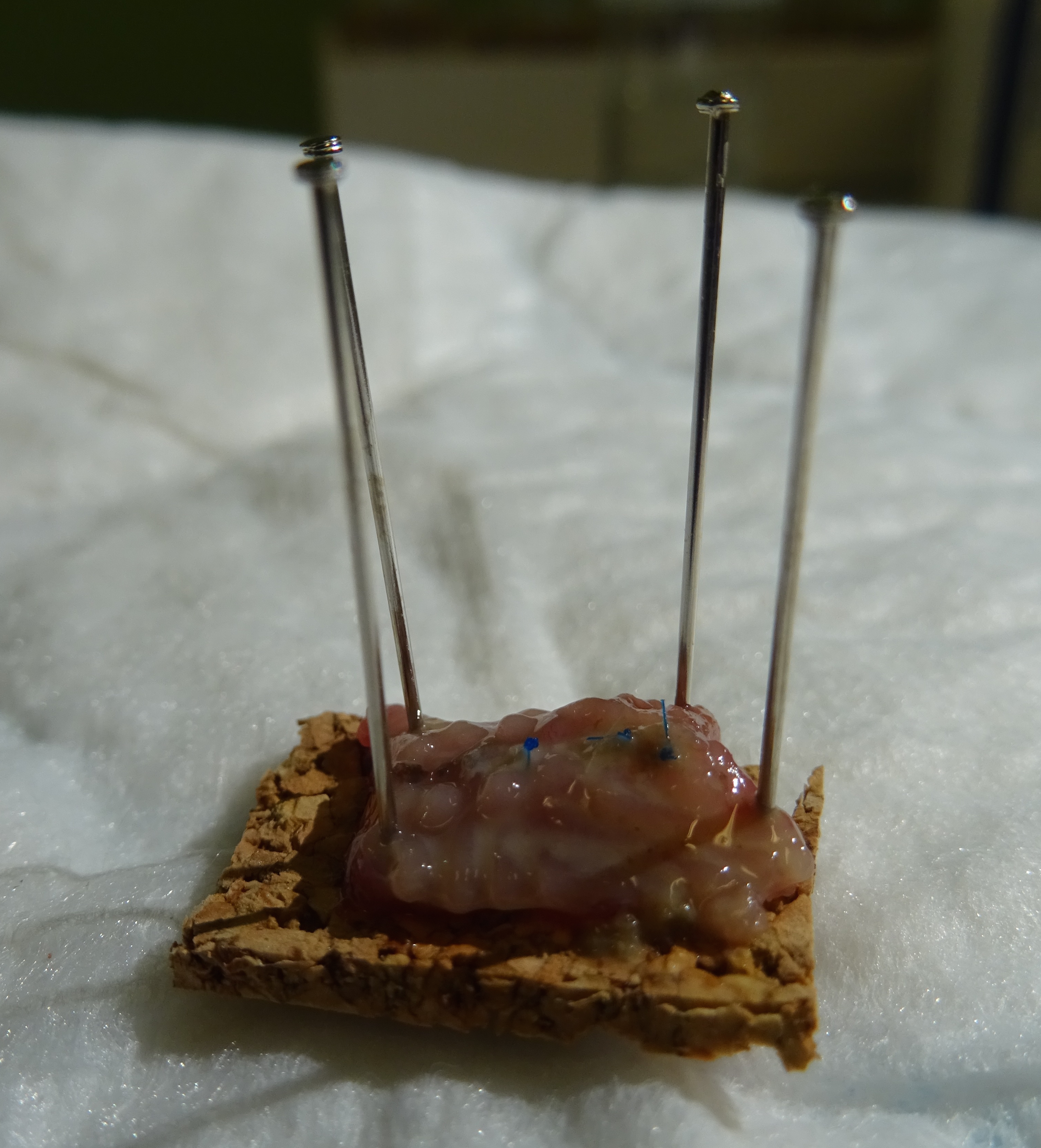


*Figure S2: One-third of the anastomotic site, obtained for immunohistochemistry purposes. Tissue was stretched and pinned onto a cork layer in order to secure a straight anastomotic line and improve quality of histological assessment prior to fixation in formalin.*
